# A Sensor System for Detection of Hull Surface Defects

**DOI:** 10.3390/s100807067

**Published:** 2010-07-26

**Authors:** Pedro Navarro, Andrés Iborra, Carlos Fernández, Pedro Sánchez, Juan Suardíaz

**Affiliations:** DSIE, Technical University of Cartagena, Campus Muralla del Mar s/n, Cartagena, E-30202 Spain; E-Mails: pedroj.navarro@upct.es (P.N.); carlos.fernandez@upct.es (C.F.); pedro.sanchez@upct.es (P.S.); juan.suardiaz@upct.es (J.S.)

**Keywords:** vision sensors, surface fault detection, ship hull blasting

## Abstract

This paper presents a sensor system for detecting defects in ship hull surfaces. The sensor was developed to enable a robotic system to perform grit blasting operations on ship hulls. To achieve this, the proposed sensor system captures images with the help of a camera and processes them in real time using a new defect detection method based on thresholding techniques. What makes this method different is its efficiency in the automatic detection of defects from images recorded in variable lighting conditions. The sensor system was tested under real conditions at a Spanish shipyard, with excellent results.

## 1. Introduction

One of the most common operations in ship maintenance is blasting, which consists in projecting a high-pressure jet of abrasive matter onto a surface to remove adherences or traces of rust. The objective of this task is to maintain hull integrity, guarantee navigational safety conditions and assure that the surface offers little resistance to the water in order to reduce fuel consumption. This can be achieved by grit blasting [1] or ultra high pressure water jetting [2]. In most cases these techniques are applied using manual or semi-automated procedures with the help of robotized devices [3]. In either case defects are detected by means of human operators; this is therefore a subjective task and hence vulnerable to cumulative operator fatigue and highly dependent on the experience of the personnel performing the task.

Various authors have recently addressed the development of sensor systems supported by the infrastructure supplied by computer vision systems. The possible fields of application are quite diverse, including automatic robot welding [4], 2-D position measuring [5], vehicle applications [6], optical measuring of objects [7], distance estimation [8] and others. Vision systems have likewise been used to detect defects on large metal surfaces. There are therefore numerous references in the literature [9] that meet sets of requirements which differ depending on the application (type of defects, volume of defects to be detected per time unit, precision, robustness, *etc*.). In systems of this kind controlled lighting systems are commonly used to highlight the defects and thus simplify the subsequent phases of image pre-processing and segmentation. However, such solutions are not acceptable in the case of inspection of large surfaces under variable and non-uniform lighting conditions as in automatic detection of surface defects in ship hulls in the open air. There is therefore a need to define a model that will make it possible to detect defects of this kind in real time with a high rate of accuracy. Such a method must be implemented in a system that is robust enough to be used in an aggressive environment such as a shipyard.

This paper proposes a sensor system for detecting defects in ship hulls which is simple enough to be implemented in such a way as to meet the real-time requirements for the application. This sensor considers a local thresholding method, which is based on the automatic calculation of a global reference value that has been denominated Histogram Range for Background Determination (HRBD). This value is subsequently used to calculate the local threshold of each area of the image, making it possible to determine whether or not a pixel belongs to a defect. The method has been tested against other classic thresholding methods and has proved highly stable in variable lighting conditions. At the same time, the proposed sensor system has been implemented and validated in real conditions at a shipyard in Spain.

Section 2 details the constituent elements of the sensor system and the sequence followed in the processing of the images captured. Section 3 details the defect detection method that has been developed. Section 4 presents the implementation of the sensor system developed for the shipyard case study, the measurements considered to assess the performance of the method, a comparison of the results with those of other common thresholding methods, and the results of the sensor system tests at the shipyard. Finally, Section 5 presents our conclusions.

## 2. Sensor System

**Figure 1.**
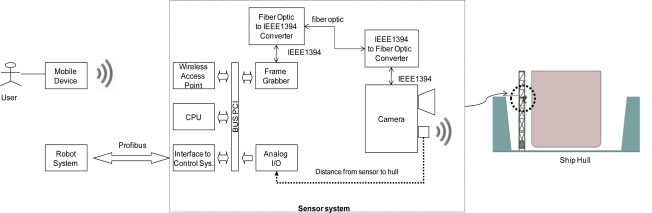
Block diagram of the sensor system.

**Figure 2.**
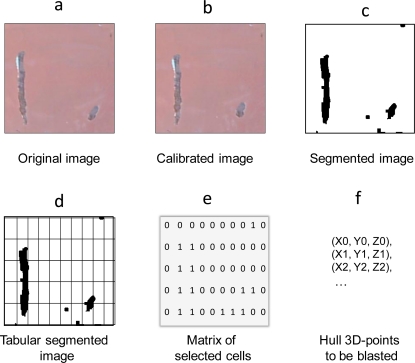
Example of image processing sequence with the proposed sensor.

The sensor system proposed in this article (Figure 1) operates on the basis of image acquisition via a digital camera equipped with a wide-angle lens. The camera is placed so that its optical axis is perpendicular to the plane of the surface to be inspected. The distance between that plane and the camera is measured with the help of an ultrasound sensor with a working range of 40 to 300 cm. The images obtained with this lens are slightly distorted (Figure 2(a)), and therefore they have to be corrected with a camera model that includes the intrinsic (focal distance, image centre, radial and tangential distortion of the lens) and extrinsic (rotation matrix and translation vector) parameters of the camera. These parameters are derived by a one-off calibration in the workshop before the sensor system is put into operation at the shipyard. The software used for this purpose was the Toolbox for Matlab Caltech [10] which is coming to be one of the commonly used calibration software. This toolbox implements, inter alia, the method developed by Zhang [11]. That method requires the camera to observe a flat pattern from several (at least two) viewpoints, so that both the camera and the flat pattern can move freely without the need to identify that movement.

Once the sensor system has been calibrated, it is ready to operate in real conditions at the shipyard. From that point on, the sensor system corrects the distorted images captured by the camera in real time (Figure 2(b)). That correction factors in the distance detected by the ultrasound sensor so that the camera model derived from the calibration procedure is loaded for each distance. The defect detection method proposed in this article is applied to the corrected image. This method makes it possible to obtain another image in which all the detected defects are marked (Figure 2(c)). The position of the defects in the image and the parameters derived from the calibration are used to find the 3D coordinates of the points on the hull that the robot has to access for cleaning.

All cleaning methods (Grit Blasting, UHP Water Jetting) are based on the projection of a jet of grit or water of a given width, and so the image is divided into cells (Figure 2(d)). The size of these cells indicates the area that the jet of grit or water is capable of cleaning when projected on to the vessel’s surface. In this way the sensor system sends back a matrix of MxN cells (Figure 2(e)) where there may be defects and grit blasting may be necessary. The cell size is a user-defined input parameter. This is calculated from the distance between the blasting nozzle and the ship hull, the speed of the grit jet and the rate of cleaning head movement.

Finally, it is important to note that the images obtained in this way at the shipyard are typically captured in the open air and under highly variable atmospheric and lighting conditions. This is an aspect that will very much influence the method that is designed for defect detection as described in the following section.

## 3. Method for Defect Detection: UBE

The proposed method for the detection of defects has been denominated UBE (thresholding based on Unsupervised Background Estimation) and has been divided into two stages. In the first stage a global calculation is carried out on the images to estimate a parameter that has been called a Histogram Range for Background Determination (HRBD). This will serve as a reference during the local calculation. In the second stage, using this parameter as a starting point, the image is binarized following the steps detailed below.

### 3.1. First Stage. Determination of HRBD and Sensitivity

**Figure 3.**
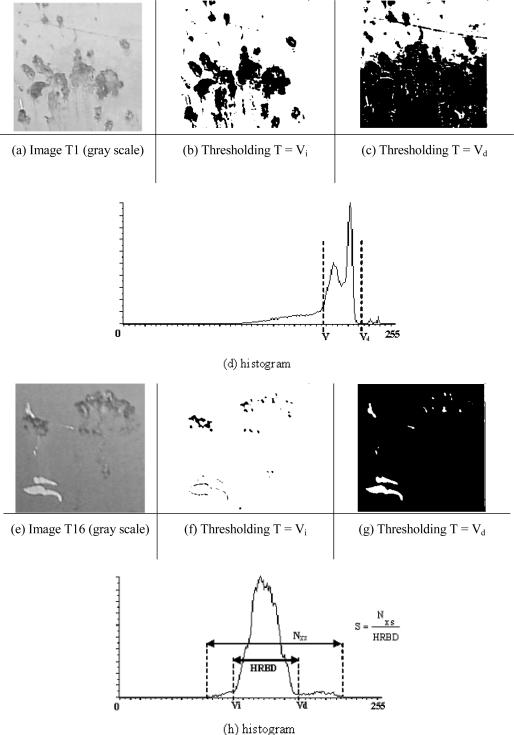
Segmentation of defects for images taken under different lighting conditions.

The proposed method is inspired by an algorithm described by Davies [12] and used in systems of document exploration for optical character recognition. Davies’ method executes the segmentation of images by determining a threshold once the percentage of existing characters with regard to the background is known. This algorithm is not suitable for application to images where it is not possible to determine the foreground/background ratio beforehand. The method proposed in this article, thresholding based on Unsupervised Background Estimation (UBE), is an improvement on the method described by Davies in that it makes it possible to automatically estimate the foreground/background ratio by analysing the histogram of the image. Figure 3 shows two typical situations that can arise with images of ship hulls taken in different conditions. The first of these (T1) was taken at the shipyard with a solar radiation level of 385 W/m^2^ and the second (T16) with a level of 252 W/m^2^. We can observe the following:

- The histograms belonging to both images present a visible maximum which corresponds to background. This always happens in the common hypothesis of a foreground/background ratio less than 1. There are also other relative maxima produced by defects (foreground), noise and/or lighting effects.
- The better the lighting of the scene, the greater is the difference between the significant maxima in the histogram. This fact can be verified in more detail in Figure 3(d), which shows two clearly differentiated maxima. If the threshold T = Vi is selected to binarize the image, it is possible to detect most of the darker defects (see Figure 3(b)). In this case the grey levels of the image corresponding to the background can be seen within the range [Vi–255]. Even so, this range includes defects whose grey level approaches white (see Figure 3(c)). The segmentation can be further improved by restricting the range rightwards up to the first valley located on the right of the maximum value of the histogram (Vd) due to the existence of defects in the range [Vd–255].
- The poorer the lighting, the more overlap there is between the grey level distributions. Figure 3(e) shows an image of defects with low, uneven illumination, in which we can observe overlapping distribution. As in the previous case most of the darker defects can be detected by means of the threshold T = Vi (see Figure 3(f)), while the clearer areas can be detected with the threshold T = Vd (see Figure 3(g)).

After performing these observations, the greater part of the background was judged to be situated between points Vi and Vd. The difference between these two values has been called the HRBD-Histogram Range for Background Determination.

Once the HRBD has been calculated, a sensitivity value (S) is calculated so that the calculation of the local threshold in the second stage of the method can be fine-tuned; this value is a ratio determined by the number of histogram entries different from zero (Nxs) divided by the HRBD (see Figure 3(h)). This value computes the ratio between the total size of the histogram with values different from zero and the estimated size of the background, HRBD, and is calculated according to the following Equation:

(1) $$S=\frac{N_{\text{xs}}}{\text{HRBD}}$$

**Figure 4.**
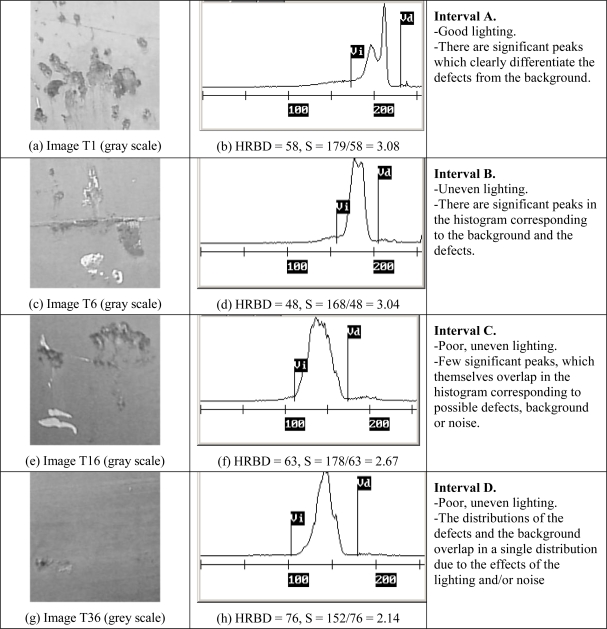
A selection of images with defects and different illumination conditions (a), (c), (e), (g), their corresponding histograms and determination of the HRBD (b), (d), (f), (h).

Figure 4(b,d,f,h) shows graphically the results of calculating the HRBD = Vd − Vi on the histograms of four images from the case study (Images T1, T6, T16 and T36). In the figures, note how the points that allow the calculation of the HRBD are located on both sides of the most significant distribution. An intensive search for the significant minima both to the left (Vi) and to the right (Vd) of the main distribution was performed to determine the HRBD automatically, in which a significant minimum was taken to be the nearest minimum to the left or right, respectively, of the maximum of the histogram. Also shown are the numeric values derived from calculation of the HRBD and the sensitivity.

### 3.2. Second Stage. Segmentation of the Image Pixels

Once the HRBD has been calculated, the image is scanned pixel by pixel to determine which pixel belongs to the background and which does not. To do this, the method analyses the neighborhood of each pixel; this neighborhood is formed by a window of size k × k centred on the pixel in question, k being a natural odd number greater than one and smaller than the dimensions of the image. The neighborhood analysis determines the value of the local threshold (t) for the k pixel binarization.

To determine the local threshold of each pixel, first the range of the neighborhood of the pixel is determined as the difference between the maximum and the minimum of the Local Grey Level (LGL):

(2) r=max LGL−min LGL

If the range is equal to or lower than the HRBD, the threshold is fixed by:

(3) t=max LGL−HRBD/S

If the range is greater than the HRBD, the threshold is calculated by:

(4) $$t=\frac{\text{max}\;\text{LGL}+\text{min}\;\text{LGL}}{2}$$

**Figure 5.**
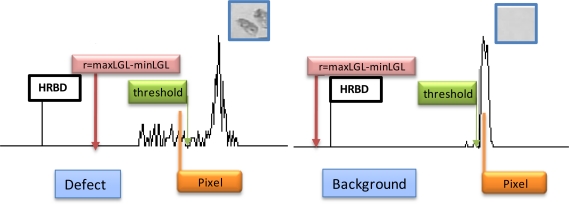
Determination of defects and background.

If the current value of the pixel is equal to or greater than this threshold it is considered to be a background pixel (grey level 255); otherwise it is considered as a pixel associated with the defect and is allotted a grey level value of zero. Figure 5 details the relationship between r and HRBD that implies when a defect is present or not.

**Figure 6.**
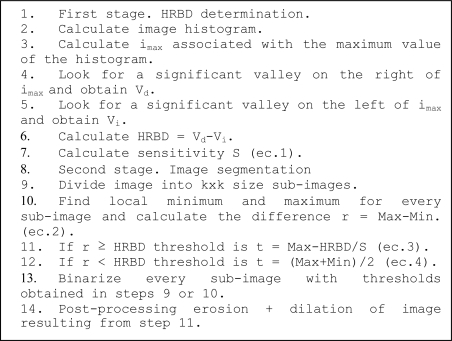
UBE algorithm.

Finally, the binarized image is processed using an erosion filter followed by a dilation filter to reduce the noise, which improves the performance for foreground (defects) segmentation. The pseudocode for the algorithm followed by the sensor system in operation is depicted in Figure 6.

## 4. Sensor System Validation

### 4.1. Sensor System Implementation

The sensor system has been implemented on a Pentium-IV at 2 GHz with a Matrox Meteor II/1394 card. This card is connected to the microprocessor via a PCI bus and is used as a frame-grabber. For that purpose the card has a processing node based on the TMS320C80 DSP from Texas Instruments and the Matrox NOA ASIC. In addition, the card has a firewire input/output bus (IEEE 1394) which enables it to control a half-inch digital colour camera (Sony DFW-SX910) equipped with a wide-angle lens (Cosmiscar H416 4, 2 mm). The software development environment used to implement the system software modules was the Visual C++ programming language powered by the Matrox Imaging Library v8.0. The system also has a Siemens CP5611 card which acts as a Profibus-DP interface for connection with the corresponding robotized blasting system. A Honeywell sensor is used to measure the distance to the ship by ultrasound, with a range of 200–2,000 mm and an output of 4–20 mA. User access to the sensor system is by means of an industrial PDS (Mobic T8 from Siemens) and a wireless access point. Among other functions, the software that has been developed allows the operator to: (1) enter the system configuration parameters, (2) visualize the possible cleaning points for validation by the operator before blasting commences, and (3) calibrate the sensor system.

### 4.2. Validation Environment

**Figure 7.**
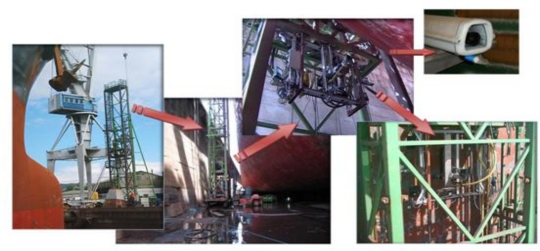
Robotized tower with secondary system mounted (XYZ table).

The proposed sensor system was assessed at the Navantia shipyard in Ferrol (Spain) on a robotized system used to perform automatic spot-blasting. This kind of blasting consists in cleaning only areas of the ship hull that are in poor condition rather than the entire hull. This operation accounts for 70% of all cleaning work carried out at that shipyard. The robotized system (Figure 7) consists of a mechanical structure divided into two parts: primary and secondary. The primary structure holds the secondary structure (XYZ table), which supports the cleaning head and the sensor system. More information regarding this system can be found in [13].

With the help of this platform, 200 images of the ship hull were taken, similar to T1 and T16 in Figure 3. In this way a catalogue was compiled of typical surface defects as they appear before grit blasting. Images were acquired at different points in time and were classified into four time intervals: A (8 a.m. to 10 a.m.), B (10 a.m. to 1 p.m.), C (1 p.m. to 4 p.m.) and D (4 p.m. to 7 p.m.). In this way it was possible to achieve a complete analysis of the sensor system’s performance, which included its behaviour in variable lighting conditions.

The images illustrate three main features of the lighting conditions that the algorithms must deal with: (1) lighting conditions vary as the sun’s position and weather conditions change in the course of the day, (2) lighting is not uniform because of the position of the hull relative to the sun, and (3) there is a difference in brightness between the upper and the lower levels of the dry-dock.

### 4.3. Metrics and Performances

In order to conduct a quantitative analysis of the quality of the proposed segmentation method as compared to other methods, we need to use the metrics best suited to that purpose. The performance of image segmentation methods has been assessed by such authors as Zhang [14], Abak [15] and Sezgin [16]. They propose different metrics to allow measurement of the quality of the segmentation in a given method, using parameters like position of the pixels, area, edges, *etc*. Of these, three of the quantitative appraisal methods proposed by Sezgin have been selected and are examined below.

#### Misclassification Error-ME

The ME error represents the percentage of the background pixels that are incorrectly allocated to the object (*i.e.*, to the foreground) or *vice versa*:

(5) $$\text{ME}=1-\frac{\left|B_{P}\cap B_{T}|+|O_{P}\cap O_{T}\right|}{\left|B_{P}|+|O_{p}\right|}$$

The error can be calculated by means of Equation 5, where BP (Background Pattern) and OP (Object Pattern) represent the pattern image of the background and of the object taken as reference, and BT (Background Test) and OT (Object Test) represent the image to be assessed. In the event that the test image coincides with the pattern image, the classification error will be zero and therefore the performance of the segmentation will be maximum.

#### Relative Foreground Area Error-RAE

The RAE error is defined by Equation 6, where A_P_ is the value of the area obtained from the pattern image and A_T_ the area of the segmented image. An optimal segmentation will produce a result of RAE equal to zero:

(6) $$\text{RAE}=\{\begin{array}{lll} \frac{A_{P}-A_{T}}{A_{P}} & \text{if} & A_{T}<A_{P}\\ \frac{A_{T}-A_{P}}{A_{T}} & \text{if} & A_{P}\leq A_{T} \end{array}$$

#### Edge Mismatch-EMM

This method assesses the discrepancies between the edges of the pattern image and the segmented image, using the following equation:

(7) $$\text{EMM}=1-\frac{\text{CE}}{\text{CE}+\varpi \sum_{k\in \{\text{EO}\}}\delta (k)+\alpha \sum_{l\in \{\text{ET}\}}\delta (k)}$$

where CE is the number of coincident pixels between the edges of the pattern image and the segmented image; EO is the excess of pixels in the pattern image with respect to the segmented image; ET is the excess of pixels on the edge of the segmented image in relation to the pattern image; ω is the coefficient of penalization associated with pixels EO; α is the coefficient that penalizes the pixels ET; and δ(k) denotes the Euclidean distance from the pixel k on the edge of the segmented image to its complementary pixel in the pattern image inside a search area determined by the maximum parameter distance.

The weighted average corresponding to each of the calculated metrics (ME, AE and EMM) is the parameter by which the global performance of the implemented algorithms is assessed according to the equation:

(8) $$\eta =100^{*}(1-\frac{\text{ME}+\text{AE}+\text{EMM}}{3})$$

### 4.4. System Sensor Appraisal

In order to check the quality of the proposed segmentation algorithm (UBE) for the sensor system, two alternative solutions were also implemented, based on two well-known classic thresholding algorithms. The first of these (Alg1) is based on the method of Otsu [17] and the second (Alg2) on the method of Niblack [18].

Otsu’s method considers “as optimal” the grey level that maximizes the variance between classes. For this purpose it considers the use of a set of theresholds. In this case study we consider two classes present in the image: the background and defects, so being only necessary the calculation of one global threshold. Niblack’s method locally adapts the threshold according to the local mean and standard deviation (calculated in windows of k × k pixels size).

**Figure 8.**
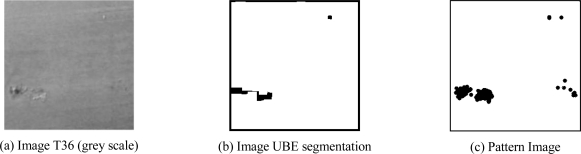
A sample of the processed images.

The three solutions were applied to the catalogue of 200 images that had been taken at the shipyard (one of these is shown in Figure 8(a)). The result was 3 × 200 images in which the defects had been segmented using each of the three methods considered (one of them is shown in Figure 8(b), corresponding to the UBE method). To apply the metrics described above, human inspectors were needed to segment each of the catalogue images manually (one of these is shown in Figure 8(c)).

Table 1 shows the results after applying each of the proposed algorithms (Alg1, Alg2 and UBE) to the catalogue of 200 images. The average values and the yield were calculated for each of the three metrics considered (ME, RAE and EMM). The average value of the three metrics and the average yield were also calculated. As the table shows, the best yields were achieved with the proposed UBE method (see shaded figures).

**Figure 9.**
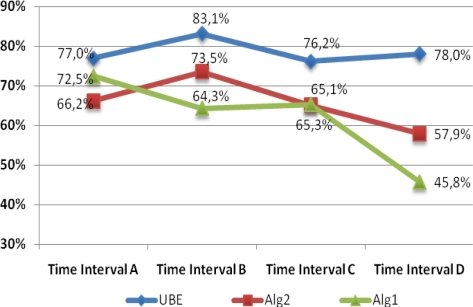
Performance progression as a function of lighting conditions.

Figure 9 shows the mean yields for each time interval (A, B, C and D). Note how the three algorithms show maximum efficiency when lighting conditions are best (time intervals A and B). As lighting conditions get worse, the performance of both Alg1 and Alg2 deteriorates rapidly compared with UBE, which maintains its performance in the worst conditions (time interval D). We may conclude that the UBE algorithm presents the best stability when faced with changes in lighting conditions.

### 4.5. Results for the Sensor System Incorporated in the Cleaning Robot

To validate the sensor system in real working conditions, it was used in the blasting of an oil tanker having a length of 120 m and a height of 12 m, using pyrite slag as grit. The target surface, with defects evenly distributed over the entire length of the hull and covering approximately 30% of the total surface, was divided into 360 panels 2 m wide by 2 m high.

**Figure 10.**
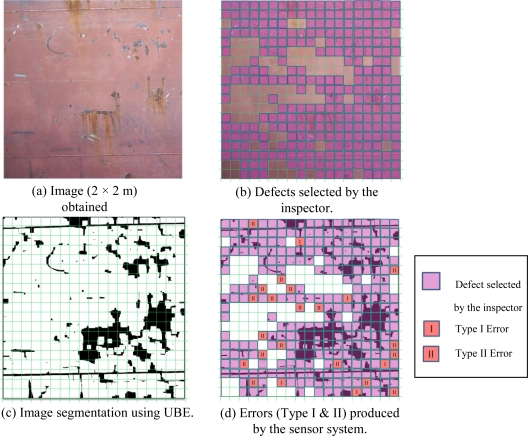
Sensor system validation process: sequence followed for a panel.

Before the tests began, an inspector examined the areas that had to be blasted in each panel, with the aid of a camera (Figure 10(a,b) shows an example of a panel). An operator then blasted half of the panels and the other half was blasted by the robotized system equipped with the proposed sensor system. Figure 10(c) shows the segmented image calculated by the sensor systems and used in the process that determined the XYZ points on the hull which contained defects.

Figure 10(d) shows the discrepancies between the calculations made by the sensor system and those of the inspector. It identifies the cells marked by the system as defective when they were not (Type I error) and the cells marked free of defects when in the inspector’s opinion they required treatment (Type II error).

The panels that had been blasted by the operators were also inspected to identify discrepancies with the inspector’s analysis. Table 2 shows the average number of cells cleaned by the operator and by the sensor system with Type I and Type II errors for the 360 panels indicated above.

As we can see, the sensor system produced better results as regards false positives—*i.e.*, cells marked as defective when they are not (Type I error). This is essentially because the operator tends to blast larger areas than are necessary, and moreover he is less able to control the cut-off of the grit jet. On the other hand, the sensor system identified more false negatives (Type II error) than the operator. This difference was not very significant and is quite acceptable in view of the clear advantages offered by the sensor system as regards Type I errors.

## 5. Conclusions

This paper has presented a sensor system based on an original thresholding method (UBE), especially suited for image segmentation under variable and non-uniform lighting conditions. A comparison of the method proposed for detection of defects with other classic thresholding methods shows that it achieves a higher performance. The sensor system incorporates a robotized system for cleaning ship hulls, making it possible to fully automate grit blasting. The results as regards to reliability were very similar to those achieved with human operators, while faster (15–25%) inspection was achieved and the consequences of operator fatigue minimized. The proposed sensor system can readily be used in other robotized cleaning systems using either grit or pressurized water.

## Figures and Tables

**Table 1.** Algorithm performance for image segmentation.

**Algorithm**		**ME**	**RAE**	**EMM**	**Average**
**Alg1**	**Metric**	0.178	0.605	0.359	0.380
	**Perform. (**η**)**	82.2%	39.5%	64.1%	62.0%
**Alg2**	**Metric**	0.135	0.323	0.572	0.343
	**Perform. (**η**)**	86.5%	67.7%	42.8%	65.7%
**UBE**	**Metric**	0.075	0.262	0.306	0.214
	**Perform. (**η**)**	92.5%	73.8%	69.4%	78.6%

**Table 2.** Comparison between human inspection and automated inspection.

**Average Data**	**Human Inspection (180 panels)**	**Automated Inspection (180 panels)**
**Type I Error**	13%	7.3%
**Type II Error**	1.8%	2.6%

## References

[1] Momber A (2008). Blast Cleaning Technologies.

[2] Le Calve P (2007). Qualification of Paint Systems after UHP Water Jetting and Understanding the Phenomenon of “Blocking” of Flash Rusting. J. Protec. Coat. Lin.

[3] Ortiz F, Alonso D, Pastor P, Alvarez B, Iborra A, Habib MK (2007). Bioinspiration in Robotics. Walking and Climbing Robots.

[4] Park J, Lee S, Lee IJ (2009). Precise 3D Lug Pose Detection Sensor for Automatic Robot Welding Using a Structured-Light Vision System. Sensors.

[5] Luna C, Lázaro J, Mazo M, Cano A (2009). Sensor for High Speed, High Precision Measurement of 2-D Positions. Sensors.

[6] Hannan M, Hussain A, Samad S (2010). System Interface for an Integrated Intelligent Safety System (ISS) for Vehicle Applications. Sensors.

[7] Chiabrando F, Chiabrando R, Piatti D, Rinaudo F (2009). Sensors for 3D Imaging: Metric Evaluation and Calibration of a CCD/CMOS Time-of-Flight Camera. Sensors.

[8] Lázaro J, Cano A, Fernández P, Luna C (2009). Sensor for Distance Estimation Using FFT of Images. Sensors.

[9] Zheng H, Kong LX, Nahavandi S (2002). Automatic Inspection of Metallic Durface Fefects Using Genetic Algorithms. J. Mater. Process. Tech.

[10] Bouguet JY Camera Calibration Toolbox for Matlab. http://www.vision.caltech.edu/bouguetj/calib_doc/.

[11] Zhang Z (2000). A Flexible New Technique for Camera Calibration. IEEE Trans. Pattern. Anal.

[12] Davies ER (2005). Machine Vision: Theory Algorithms Practicalities.

[13] Iborra A, Pastor J, Alonso D, Alvarez B, Ortiz FJ, Navarro PJ, Fernández C, Suardiaz J (2010). A Cost-Effective Robotic Solution for the Cleaning of Ships’ Hulls. Robotica.

[14] Zhang YJ A Review of Recent Evaluation Methods for Image Segmentation.

[15] Abak AT, Baris U, Sankur B The Performance Evaluation of Thresholding Algorithms for Optical Character Recognition.

[16] Sezgin M, Sankur B (2004). Survey over Image Thresholding Techniques and Quantitative Evaluation. J. Electron. Imaging.

[17] Otsu NA (1978). Threshold Selection Method from Gray-Level Histograms. IEEE Trans. Syst. Man. Cybren.

[18] Niblack W (1986). An Introduction to Digital Image Processing.

